# Online Store Aesthetics Impact Efficacy of Product Recommendations and Highlighting

**DOI:** 10.3390/s22239186

**Published:** 2022-11-26

**Authors:** Piotr Sulikowski, Michał Kucznerowicz, Iwona Bąk, Andrzej Romanowski, Tomasz Zdziebko

**Affiliations:** 1 Department of Information Systems Engineering, Faculty of Information Technology and Computer Science, West Pomeranian University of Technology, ul. Zolnierska 49, 71-210 Szczecin, Poland; 2 Department of Applied Mathematics in Economics, Faculty of Economics, West Pomeranian University of Technology, ul. Zolnierska 47, 71-210 Szczecin, Poland; 3 Institute of Applied Computer Science, Faculty of Electrical, Electronic, Computer and Control Engineering, Lodz University of Technology, ul. Stefanowskiego 18/22, 90-924 Lodz, Poland; 4 Department of Information Technology in Management, Faculty of Economics, Finance and Management, University of Szczecin, ul. Adama Mickiewicza 64, 71-101 Szczecin, Poland

**Keywords:** eye tracking, aesthetics, user attention, recommendations, highlights, e-commerce, user experience, human–computer interaction

## Abstract

Owing to high competition in e-commerce, customers may prefer sites that ensure that good user experience (UX) and website aesthetics are one of its qualities. The method of presenting items seems crucial for gaining and maintaining user attention. We conducted a task-based user eye-tracking study with *n* = 30 participants to examine two variants of an online fashion store: one based on aesthetic rules and one defying them. The following aspects of item presentation were considered: height and width the ratio of product photos, website colors, rounded borders, text visibility, spacing between elements, and smooth animation. We investigated their relationship to user attention by analyzing gaze fixation, tracking user interest, and conducting a supplementary survey. Experimental results showed that owing to following the rules of aesthetics in interface design in the presented fashion shopping scenario, elements such as the recommendation area and product highlights had a significant positive impact on customer attention.

## 1. Introduction

The growth of the Internet is connected with an increase in e-commerce activity [1], and thus competition and the need to attract customer attention. It is good when a website remains in the customer’s memory, stands out in a certain way, and at the same time is aesthetic and useful. Therefore, user experience (UX) design plays an increasingly important role [2]. To ensure refined aesthetics, it is necessary to know the meaning of specific elements of the online store to the user.

The most important element from the user’s perspective is the product itself [3], which is why we focus on how the product is presented. Leaving aside factors such as the choice of words or the price, there is a large range of aesthetic features that can affect the user’s perception of the store. One of the basic parameters is the proportion of the height and width of photographs showing the product. These proportions may, for example, follow the golden ratio, a value found in nature and considered to represent ideal proportions [4]. In addition, the way the item is presented in the product picture seems to be important [5]; for example, in the case of clothes, whether they are visible on the model or maybe just on their own. Following this, for clothes on models, the model’s position may be important, whether they are relatively static and the model is fully visible, or perhaps a dynamic standout position would be better. Another aspect is the overall consistency of elements on the webpage or any disturbance to such consistency [6]. An important factor influencing users’ attention may also be special product highlights, such as an additional visual border or an indicator informing them about the uniqueness of the product in comparison with others.

For visual perception of the store, the overall aesthetics are an important issue, in particular so that the products are clearly visible and easily perceivable by the customer. An important aspect of that is the colors of the page, which should refer to the purpose of the page and not overwhelm the user [7], who should be able to orient themselves on the page easily and willingly return to it. It is also worth mentioning the distances between the elements on the page so that the whole page is consistent, for example in accordance with the Gestalt proximity principle [8].

This paper is an extension of the research to date on the impact of e-commerce website aesthetics on customer attention attraction in terms of specific, commonly employed elements. Specifically, this work contributes with a comparative examination of online shopping user experience for a purpose-built online fashion store deployed in two versions: aesthetic and unsightly (non-aesthetic). There are only a few studies comparing such variants of a website, and our study exploits this research gap [9,10,11]. It is based on a study utilizing eye and mouse/event tracking and a supplementary survey. The rest of the paper is structured as follows: related work is presented in Section 2, experimental design is presented in Section 3, and results are presented in Section 4.

## 2. Related Work

There are three dimensions of visual design quality: aesthetic, functional, and symbolic. Recent studies suggest that all three qualities positively influence the intention to use a website and positive word of mouth, and that website aesthetic quality positively influences website functionality and symbolic qualities [12]. Kivijärvi et al. [13] demonstrated that usability strongly correlated with a satisfactory user experience. Other research has shown that the appearance of a website is more important than its usefulness in terms of its impact on user satisfaction [7] and suggested that UI designers would be well-advised to create aesthetically appealing sites that clearly and immediately reflect their purpose. In particular, it was discovered that two web design attributes—navigation bar design and performance—were the main predictors of perceived usability [14]. However, there are also recent reports suggesting that aesthetics may have little or no impact on the performance of tasks on a website [9], as well as on the user’s perception of website usability [10].

Some studies focus on the most important aesthetic features that affect users. Ngo and Byrne [11] suggest that by customizing, inter alia, the shapes and sizes of page elements one can achieve a balanced user experience thanks to a balanced composition that does not overwhelm the viewer. In another paper [6], they present 14 aesthetic features most influencing users, including numerous possible proportions of the elements on the site: square (1:1), square root of two (1:1.414), golden rectangle (1:1.618), square root of three (1:1.732), and double square (1:2). This study indicated that the 14 features are important determinants of system acceptability, understood as the correlation between interface aesthetics and usability. Many studies focused on the size of banners. Baltas [15], in examining the influence of banner characteristics on user response, found that a larger banner size (in pixels) has a better impact on the user, attracting their attention and triggering positive reactions. Similarly, Robinson et al. [16] have shown that larger size increases the effectiveness of banners. An interesting though rarely discussed aspect of website aesthetics is the golden ratio. For example, Namin et al. [17] showed that banners with dimensions similar to the golden ratio significantly increase user engagement through the number of clicks. Another study [18] also describes the influence of the golden ratio on the aesthetic evaluation of the product, but there was no definite conclusion. It was hypothesized that the golden ratio may have a positive effect on aesthetic evaluation, but dependent on the context and other aesthetic forms used.

A substantial body of research to date has focused on the influence of the general aesthetics of websites on their perception by the user. Pappas et al. [19] showed that the behavior of the client’s gaze is related to the perception of visual aesthetics. Another study [20] comparing different types of approaches included assessments measuring the user’s perception of visual aesthetics in recent years. Palmer et al. [21] claimed that the assessment of visual aesthetics is highly subjective. Seckler et al. [22] investigated the user’s assessment of aesthetics by combining objective and subjective factors. In another study, Ismail [23] discovered that unattractive interfaces confuse, obscure intent, and slow down users’ actions. Soui et al. [24] emphasized that the interface design, apart from following the general rules, should be meaningful to the user. The aesthetics of user interfaces was widely discussed by Schlatter and Levinson [25], who reported that the interface as well as order and consistency of the grouping of elements has a significant influence on user perception. In addition, they indicated that leaving white space within gaps between elements helps to distinguish these elements of the website, and the visual design may affect the effectiveness of the users, avoiding mistakes and improving their task performance. A study by Reinecke et al. [26] showed that page colors evoke many different emotions and significantly affect the aesthetics. Taking into account the state of related research considered in this section, we decided to explore how specific aesthetic aspects may influence the attention of online customers. Hence, we proposed a comparative task-based user study to track the level of attention while performing actions during online shopping.

The data necessary to make inferences concerning user interaction can be successfully collected in many ways, in particular by observing mouse cursor movement tracking and time spent on a website [27,28,29] or eye tracking and document object model (DOM) implicit event tracking [30,31].

## 3. Experimental Design

### 3.1. Methodology: Participants, Procedure, and Experimental Setup

In our study, we decided to compare the performance of aesthetic and unsightly versions of an e-commerce store and to combine eye tracking, mouse/event tracking, and a supplementary survey as methods of data collection. We had *n* = 30 participants aged 19–25 (avg. = 22.2 ± 1.6), out of whom 26 were male and 4 were female.

The eye-tracking study was performed with the use of 120 Hz Tobii Pro X3-120 research-grade eye tracker sensors mounted to the monitor. The software used for subsequent analysis of the collected data was Tobii Pro Lab, version 1.162.32461. Equipment used was from Tobii AB, which is a Swedish high-technology company that develops complete solutions for behavior research. It provides a visual user interface and dedicated software features that support research through all phases of an eye tracking experiment from test design and recording to analysis. The monitor screen used was an AOC 24” LCD (I2490VXQ) with a resolution of 1920 × 1080 pixels. Measurements from mouse/event tracking were collected in a database on the Firebase platform, and the survey was performed online using Google Forms software.

**Figure 1:**
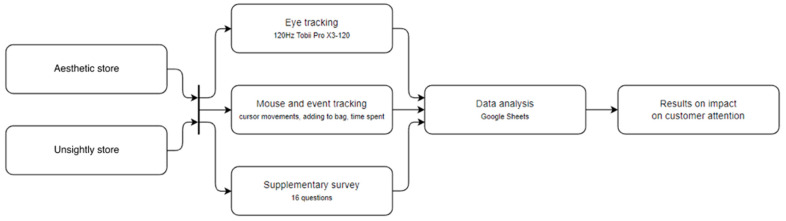
Experimental research procedure. The far left-hand column represents two experimental conditions, the column second from the left represents data acquisition methods, the third column shows the data analysis module, and the far-right column indicates the inferred results module.

First, the subjects were presented with the task to perform, and then asked to undertake the calibration of the eye tracker. After correct calibration, each participant was transferred to the first website. Males accessed the shop department with products for men, and females accessed the products for women. Study participants started either with an aesthetics-based store (called ‘aesthetic store’) or a store that broke the aesthetic principles (‘unsightly store’). The choice of the store type first shown to the user was random. In that first store, the task was to choose 1 item in each of 3 available product categories. After the selection, the participant was expected to go to the shopping cart and approve their choice. After a short break, the participant was asked to repeat eye-tracker calibration and visit the second store. The task there was the same as in the first store. It is worth mentioning that the participant was not in any way limited in time and at any moment made his own choice and moved between categories. After completing the task in the second store, the participant was transferred to a survey consisting of 15 questions. The eyesight of a participant was monitored throughout their use of the shopping websites. Figure 1 shows a scheme of the applied research procedure.

### 3.2. Store Design

**Figure 2:**
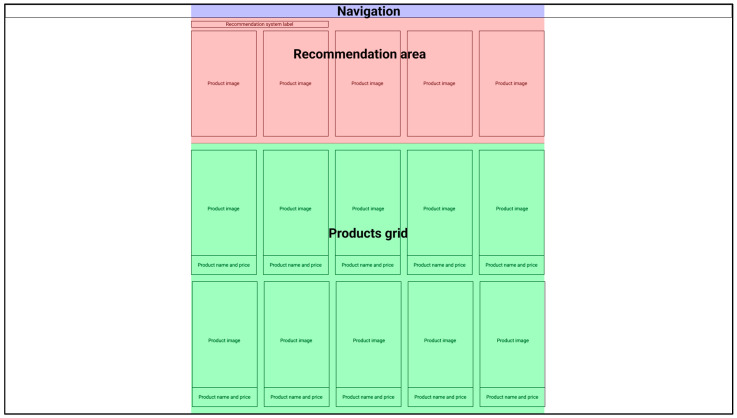
Experimental design of the online store webpage. Main subareas of the webpage highlighted in colors: Navigation (purple bar at the top), recommendation area (first below the top navigation bar), product grid (bottom green area), and background (left and right white space).

In the experiment we examined two purpose-built fashion stores, one based on aesthetic rules and one defying them. Moreover, the stores were available either with products for men or women. Each store consisted of 4 subpages: 3 product category pages and a shopping cart. The examined pages were the category pages, where one could distinguish the navigation area, including the store’s logotype and the cart icon, the recommendation interface area, the main list of products in the category, and website background (Figure 2).

The product categories included in the study were: hoodies, trousers, and t-shirts for the male store and dresses, skirts, and t-shirts for the female store. In each category, the recommendation interface offered 5 items in one row, while the main list of products consisted of 10 products in a grid of 2 rows and 5 columns. The elements in the recommendation area were static, while in the main list of products they were dynamic so that while hovering the cursor over the product a different photo of the product appeared.

**Figure 3:**
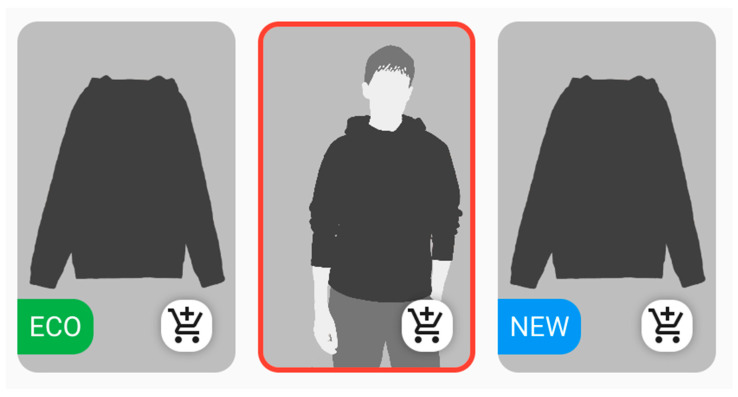
Product highlight types used. (The pictures of clothes and models are illustrative).

Additionally, the products had randomly assigned visual highlights expected to influence user perception, namely: a red border, an ‘eco’ indicator, or a novelty indicator. All of the highlight types used are presented in Figure 3. The red border was visible around the photo of the product and did not inform the user about anything specific, it was only visually different. The ‘eco’ indicator was a green element with the word ‘ECO’ in the bottom left-hand corner of the product image. The last highlight was the novelty indicator, presented as a blue element with the word ‘NEW’ in the bottom left-hand corner of the photo presenting the product.

**Figure 4:**
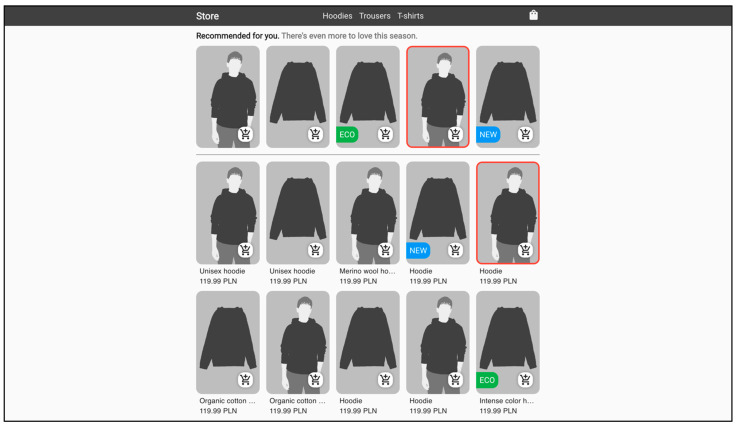
Aesthetic store’s website appearance. Color palette: modern gray and white palette, single-color background. Product photos: height-to-width ratio 1.618 (golden ratio), modern-looking rounded corners, smooth animation after hovering over with mouse cursor. Text: readable font colors, very dark gray on very light gray and vice versa. Distance between elements: short distances consistent with the rest of the interface. (The pictures of clothes and models are illustrative).

**Figure 5:**
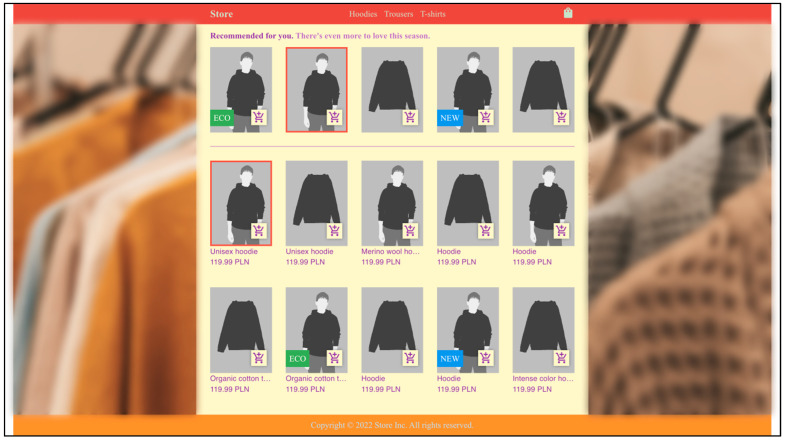
Unsightly store’s website appearance. Color palette: distinctive multi-color palette with a multi-colored image as the background. Product photos: height-to-width ratio 1.382, angular corners, abrupt change of photo on hovering over with mouse cursor. Text: contrasting, unreadable font colors: green on red, purple on yellow, and blue on orange. Distance between elements: increased distances inconsistent with the rest of the interface. (The pictures of clothes and models are illustrative).

While building the variants of the store for the experiment, the following aspects of item presentation and aesthetics were considered: the ratio of the height and width of product photos, website colors, rounded borders, text visibility, spacing between elements, and smooth animation, in order to study their relationship with user fixations and user interest. Figure 4 and Figure 5 show one of the category views in each of the two stores. One can quickly notice that the two sites differ in terms of colors and backgrounds. The aesthetic store had a modern gray and white color palette and a single-color background, while the unsightly store had a distinctive multi-color color palette with a multi-colored image as the background. Another difference was the product view design, including the photo features. In the aesthetic store, modern-looking rounded corners were used to design the view of the product, while in the unsightly store everything was angular. In addition, the height-to-width ratios of the product images differed between the pages. In the aesthetic store, the coefficient of 1.618 was used, i.e., the golden ratio, while in the unsightly store no recommended ratio was used, but rather an ad-hoc ratio of 1.382. Another aspect that distinguished both sites was text visibility. In the aesthetic store, readable font colors were used: very dark gray on very light gray and vice versa, while in the unsightly store they were contrasting, unreadable font colors: green on red, purple on yellow, and blue on orange. Another difference between the sites was the distance between the elements. Short distances were used in the aesthetic store to maintain the consistency of the entire user interface. In contrast to that, in the unsightly store greater distances were used, which seemed even greater due to the fact that the photos on this page were smaller; the justification for that was to cause a disturbance in the consistency of the interface to the customer. The last aspect that distinguished both stores, which cannot be seen in the figures below, was the animation of the products after hovering over them with the mouse cursor. In the aesthetic store, smooth animation was used to alternate the photo with another one, while in the unsightly store, the photo changed abruptly, without animation.

### 3.3. Data Collection and Analysis

Three sources of data were acquired during the study, as indicated in Figure 1. The main method of data collection was tracking eye movements of a participant browsing the online stores. Data were collected on the time and number of fixations in the selected areas of interest (AOIs). The main AOIs were the general areas of the page: navigation, product list, and recommendation interface area. There were also more specific AOIs applied, connected to the highlighting of products: a red border, a novelty indicator, or an ‘eco’ indicator.

Another data acquisition method used was based on mouse/event tracking, mainly by measuring the time the cursor was present on each of the products and by measuring all the time the participant spent in a given store.

The last data collection method in the experiment was a supplementary questionnaire, which was filled out by each participant after completing the study session. It consisted of 15 questions, among which several groups can be distinguished: demographic questions, questions about how and how much the user uses online stores, and detailed questions about the observed differences between the two online stores visited. The survey included single-choice questions, open-ended questions, and a 5-point Likert scale. The three data sources presented above complement each other in our study.

Here are the questions from the survey:

1. Age
2. Gender
3. Academic major
4. How often do you shop online?
5. Which device do you most frequently use for online shopping: computer/laptop, smartphone, or tablet?
6. Which website variant did you like more?
7. List the differences that you have noticed between the websites.
8. What influences the way you perceive the website the most: ease of use or aesthetic appearance?
9. What element of the appearance of the first store’s website aroused your greatest interest?
10. What element of the appearance of the second store’s website aroused your greatest interest?
11. Which highlight, in your opinion, had the best impact on the reception of the product: ‘new’, ‘eco’, or the red border?
12. How important is a picture of the product to you?
13. Which product picture is better in your opinion: showing a model or showing just the product?
14. During which visit to the online store did you purchase a product?
15. Do you think the featured products section influenced your selection?

First, the data obtained with the eye tracker were analyzed. They were exported to a spreadsheet file and presented in a form of readable tables. Then the data were selected and with the use of Google Sheets they were processed into a form from which one could grasp such results as average values or percentages.

The next step was to analyze the data collected from mouse movements and time spent on the sites. Initially, they were exported from Firebase to JSON, then converted to a spreadsheet file and analyzed in the same way as the eye-tracking data.

The last step was to analyze the data from the supplementary survey. Thanks to the use of Google Forms software, aggregated data with graphs illustrating the answers to the questions were available immediately after the respondents filled them in. Their answers were also exported to a spreadsheet file and analyzed statistically. Moreover, the data were cleaned or codified, especially open responses, so that as much information as possible could be extracted.

## 4. Results

### 4.1. Data Collected Using the Eye Tracker

The analysis of eye-tracking data shows that the average time spent on a website (TCT—task completion time) was 2.23 ± 0.87 min. This translates to exactly 2.22 ± 0.69 and 2.25 ± 0.87 min spent on average in the aesthetic and the unsightly store, respectively. Consequently, no significant difference between the pages was found in the average browsing times.

Table 1 shows the exact distribution of fixation time on selected elements of product category subpages. It can be observed that in the aesthetic store, the recommendation area was viewed for a longer time, as much as 34.6% of the total time spent in category subpages in that store, compared to 29% in the case of the unsightly website. Correspondingly, it can be noticed that in the unsightly store, navigation was viewed for a longer time, i.e., 6.8% compared to only 6% of the time in the aesthetic store.

**Figure 6:**
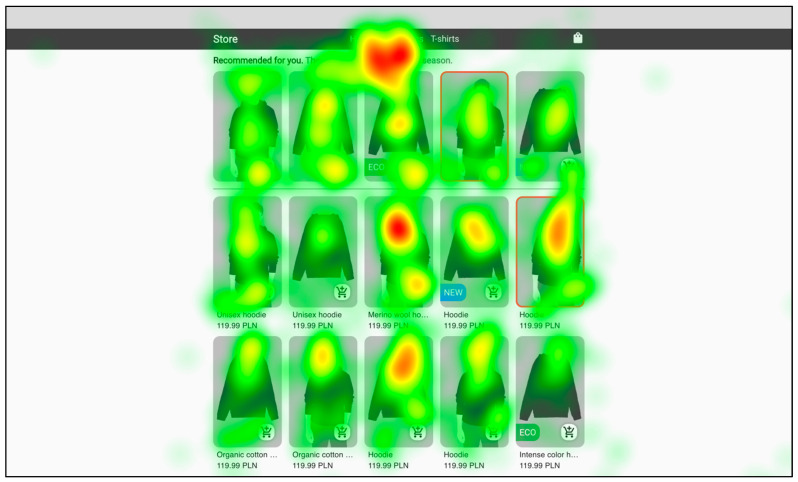
Aggregated heatmap for all participants for the first category (hoodies or skirts) in the aesthetic store. (The pictures of clothes and models are illustrative).

**Figure 7:**
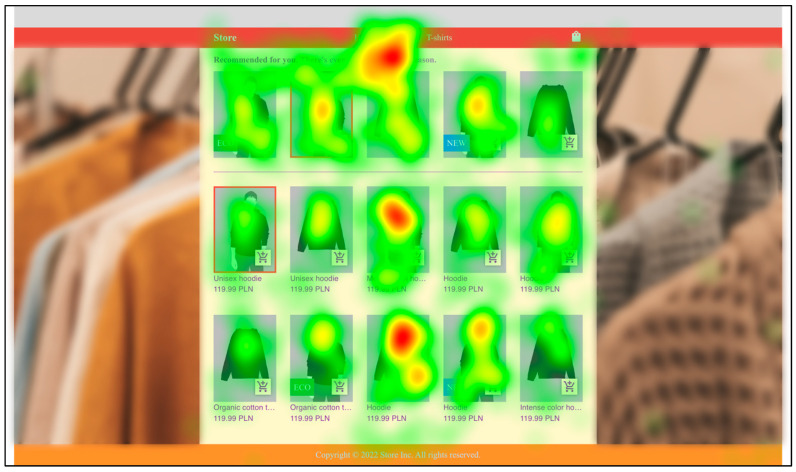
Aggregated heatmap of all participants for the first category (hoodies or skirts) in the unsightly store. (The pictures of clothes and models are illustrative).

The heatmap of the first category view (hoodies or skirts) that the user entered for all users, shown in Figure 6, clearly shows that the users’ eyesight was most concentrated in the central part of the aesthetic online store. A similar distribution can be seen in Figure 7, which also shows the heatmap of the view of the first category that the user entered for all users, but this time in the unsightly online store. Therefore, the aesthetics of the website had no apparent influence on this distribution.

From the analysis of fixation times it was calculated what proportion of the total fixation time for all products, on average, the user looked at the products with individual highlights. Since 15 products are displayed in each category view, assuming an equal distribution of fixation time across all photos, the average expected fixation value per photo would be 6.67% of the total time for all products. On average, in the aesthetic store, the products with highlights obtained 7.04% of the total fixation time on all products, while the products without any highlights gained 6.42% of the total fixation time on all products. In the unsightly store, the results were opposite: highlighted products gained less attention than non-highlighted ones, 5.93% and 7.16% of the total fixation time on all products, respectively. Detailed results are presented in the Table 2. It is worth noting that the biggest difference was observed for the red border. In the aesthetic store, products with that border achieved an average of 7.97% of the total fixation time on products, while in the unsightly store it was only 5.79%.

### 4.2. Data from Mouse and Event Tracking

After testing the choices of 174 products by the study participants, as many as 125 of them were products with the longest hover-event time compared to other products in a given category from which the user was making their choice. More precisely, the 125 products translate to 64 products in the aesthetic store and 61 in the unsightly store. As many as 72% of the products finally selected are those on which the user spent the most time with the cursor. We found interesting results by delving into the differences between the recommendation interface and the list of products while investigating the influence of the website aesthetics. In the aesthetic store, users chose 17 products with the longest hover-event time from the recommendation area, while in the unsightly store only 8 such products from the recommendation area were selected.

### 4.3. Data from the Supplementary Survey

**Figure 8:**
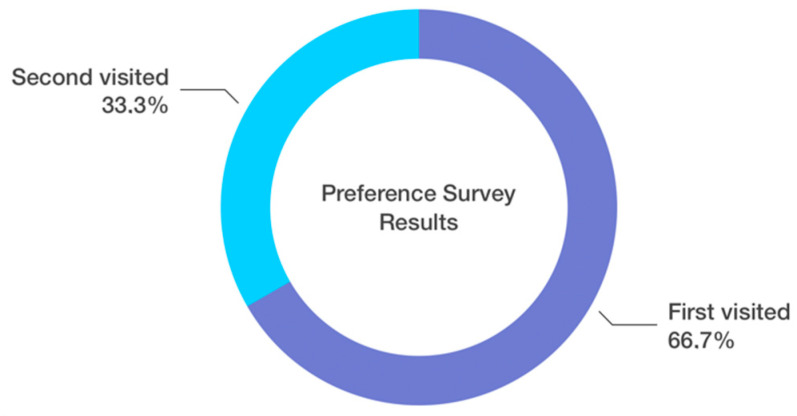
Preference survey results—responses broken down by share between the two versions of the online store.

After analyzing the responses from the supplementary survey, it appears that the division between people for whom ease of use is more important and people for whom the aesthetic appearance of the site is more important was exactly half and half. Most respondents (43.3%) admitted that they usually made purchases after several visits to a website, every third respondent needed two visits, and every fifth only one visit. Another interesting point is that when asked how important the selection of a product photo is, everyone had a positive view, meaning they replied that it was ‘definitely important’ or ‘rather important’, and no one responded ‘I have no opinion’, ‘rather unimportant’ or ‘definitely unimportant’. It should be noted that 57% of respondents preferred the photo of the product visible on the model. Another interesting result was the responses to the question of which site the user liked more. It turns out that the majority (66.7%) of the respondents replied that it was usually the first page, as shown in Figure 8, even though the aesthetic site was shown first only in 56% of instances.

**Figure 9:**
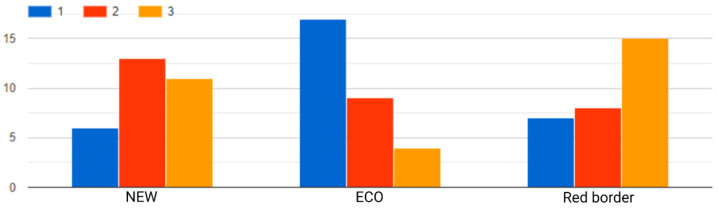
Highlight type impact survey results. Histogram of responses broken down by the impact on product perception. Scores were assigned from 1 point (smallest impact) up to 3 points (greatest impact).

In addition, the participants were asked which highlights, in their opinion, had the best impact on their perception of the products, rated from 1 to 3, with 3 meaning the greatest impact. The analysis showed that the red border declaratively had the greatest impact, in terms of the average number of the points obtained by each highlight (Table 3). In addition, as many as 50% of participants gave the red frame the maximum rating of 3 points. The data are presented in detail in Figure 9.

After analyzing the answers to open-ended questions in the survey, a number of plausible dependencies were observed. When asked about the differences between the two shops visited, the participants most often mentioned different colors, and there were also answers about highlights and navigation. In detail, 16 participants opted for the colors of the online stores, 8 indicated the overall design, 3 mentioned the highlights, 2 mentioned the background of the website, 2 mentioned the navigation bar, and yet another one pointed to site transparency (understood as the ease of browsing clothes) and a better overview of the products. Interestingly, two participants indicated the existence of the recommendation area supposedly only in the aesthetic store as the difference in stores, although it was also a part of the unsightly store. The responses to the question of which element of the website the examined subject liked the most are also noteworthy. It turns out that every third respondent did not notice or remember any elements of the appearance. The others mentioned a few items, but apart from answers similar to those as in the question on differences, the answer ‘red border’ also appeared, indicated directly by four participants, which is in line with the fixation results (the red border attracted the most user attention). Other users mentioned the highlights in general without indicating a specific one. In addition, the navigation bar was mentioned by three participants as the element which aroused the most interest, which was unexpected.

Based on the obtained survey results, it was decided to identify groups of respondents with similar views on e-commerce website aesthetics. A tool enabling this type of research is correspondence analysis, which belongs to the group of multivariate interdependence study techniques. It is widely discussed and used in socioeconomic research [32,33,34]. This method makes it possible to identify the relationships between variable variants.

We took all questions and categories of answers into account. The use of multidimensional correspondence analysis made it possible to identify three groups of respondents. The first group was constituted by women and men who make purchases once a week during their first visit to an online store. The selection of photos showing the product is definitely important for them, and they prefer photos showing a model dressed in the product. According to them, aesthetics have the greatest impact on the perception of a website. In their opinion, product distinctions affect the reception of a product in a diversified way: the new product highlights have a large impact, the red frame has a medium impact, and the distinction of an ‘eco’ product has a small impact.

The second group was women who on average make purchases once a month, after several visits to the online store, using their smartphone. They liked the first website and the aesthetic shop much more. In their opinion, the distinction of an ‘eco’ product has a large impact on the reception of the product, while the new product highlight has a medium impact, and a red frame has a small impact.

The third group were men who make purchases over the Internet less frequently than once a month, using a computer (laptop). They usually buy during the second, and sometimes even after a dozen or so visits to the store. They prefer photos of the product itself, and in our study, they looked first at the unsightly store. They believe that the perception of a product is largely influenced by the red frame highlight, while there is only a medium impact of the ‘eco’ distinction, and a small one of the novelty indicator.

## 5. Discussion

Based on the analysis of the results presented above, it is possible to summarize the impact of website aesthetics on the attention of customers in our experiment. The eye-tracking data show that the time spent on the website did not depend on its aesthetics, which is in line with the results of other recent studies [9], but activity time organization differed between the aesthetic and the unsightly shop variant. In general, on the basis of the heatmaps, it was concluded that the users’ eyesight was focused on the central part of the online store (the same as found by Djamasbi et al. [35] for browsing), and additionally it did not seem dependent on the aesthetics, as the obtained results for both shop versions were similar. It can be concluded that it is best to design the interface in such a way that the most important elements, those that one wants to draw the customer’s attention to, be located in the central part of the website.

Interesting results were obtained after analyzing fixation times for individual elements of both stores. There were significant differences in the share of fixations on main areas of the product category pages (the navigation bar, the recommendation area, and the main list of products) in the total fixation time, depending on whether the page was aesthetic or not, unlike what the latest research shows [10], but this is consistent with older studies [7]. A longer time spent looking at the navigation bar in the unsightly store may suggest that it was difficult for the user to switch between subpages, which may mean that the lack of aesthetics reduced the usability/navigation ability of the website, which contradicts some recent research [9]. It was also observed that the recommendation area was followed for a longer percentage of time in the aesthetic store. This may suggest that in an aesthetic environment it is possible to better display the store elements that we care about, thus drawing more attention to them, which is in line with previous research [23].

As for highlighting products with the red border, ‘new’, or ‘eco’ labels vs. non-highlighted items, there were observed differences in the average percentage of fixation time on a given product in relation to all products, depending on whether it was the aesthetic or the unsightly store. The highlighted products obtained a higher percentage of fixation in the aesthetic store than in the unsightly one. It is particularly noteworthy that products with the red border obtained the highest average percentage of user fixation time out of all products. It can therefore be concluded that visually highlighting a product can visibly affect the customer, especially in an aesthetic environment. On the other hand, the ‘eco’ highlight performed worse than products without any highlight. Therefore, the design of selected highlights seems to be of high importance, and these results suggest the need to conduct further research on how to adjust product features to the individual preferences of the user.

The data collected through the supplementary questionnaire showed that the study participants seemed to treat aesthetics and functionality equally, so these factors appear to be as important as shown in other studies [13]. Three clear groups of participants have been identified, showing, among others, differences in website aesthetics and product highlight perception by men and women participating in the study. An important suggestion to web designers may be to keep the composition balanced when designing a website. It was also found that when it comes to online stores, the photo showing the product is very important, not only the aesthetics around it, as other research has previously reported [5].

Referring to the highlights used on the website, it was the red border that had the greatest declarative impact on the respondents, which may suggest that such a general, somewhat mysterious highlight may attract more attention than meaningful labels such as ‘new’ or ‘eco’.

Another aspect was how users felt after using both sites. The unsightly, inconsistent colors did not seem to be a problem, but subjective perception or color tastes may have been important, which is consistent with previous research [21,22]. Despite the general claim that a site was created aesthetically or not, a particular customer may have a different, unusual taste and not fit into the framework. Therefore, in the future it might be worth focusing on the individual appearance of the stores, tailoring them to each user and their subjective preferences. An interesting result of the study is also the fact that participants mentioned navigation as a significant difference between the sites visited. This may suggest that the readability of the navigation bar is of high importance for customers and definitely makes it easier for them to navigate the online store, which is in line with previous research [14]. There were words of frustration from the respondents about the illegibility of the navigation bar in the unsightly website.

It is particularly worth emphasizing that participants paid more attention to recommendations and highlights on the aesthetic page than on the unsightly one. This may suggest that a coherent appearance allows users to see more elements, while a page that is too variegated blends into a whole. Therefore, it is worth refining the aesthetics of the interface to be able to provide customers with precisely the elements that we care about the most. This is in line with previously described principles of visual usability [25].

On the other hand, most people liked the first page they came to, regardless of whether it was aesthetic or not. This may be explained by primacy effect, a bias towards the first object considered, where information presented first has a greater effect than information presented later [36,37]. It can be concluded that it may be important for the store owner that the customer visits their store first (the first, the better), and its appearance should be remembered by them, while later-visited stores will be compared to it.

Based on the data collected from mouse movements, it can be concluded that most people chose the product on which they spent the most time with the cursor, similar to the results of other studies [29]. The most interesting results were obtained when comparing the effectiveness of the recommendation engine interface performance on the aesthetic site and on the non-aesthetic one. For the aesthetic site, products on which the user spent the most time as given by their mouse cursor movement analysis were added to the cart twice as often as for the unsightly one. The recommendation area turned out to be much more visible and effective when presented both in an aesthetic way and in an aesthetic setting, hence again confirming previous research [25]. These results also suggest that a broader examination of the correlation of the time of the mouse hover event with the final product selection by the customer would be worthwhile.

## 6. Conclusions

The growing interest in e-commerce increases competitiveness, therefore it is more and more important to attract customers’ attention by improving the aesthetics and visual usability of online stores. Results of this study, based on the analysis of data collected using eye tracking, mouse tracking, and a supplementary survey, revealed a significant impact of the website’s aesthetics on the user’s attention.

Several major conclusions have been made. While the time spent on the website did not depend on its aesthetics, the organization of the participants’ active time differed significantly between the aesthetic and the unsightly website when performing the same tasks. More attention was paid by the users to recommendations and highlights when performing tasks in the more aesthetic store, even though those elements appeared in the same manner in both stores. Moreover, the recommendation area was observed for a longer fraction of user time spent in the aesthetic store mode. This suggests that the aesthetic design helps users to notice the essential (from the perspective of sales enhancement) elements of the website. Overall, the highlighted products in the aesthetic store were given more users’ attention as expressed by the higher percentage of fixation than in the unsightly store. Products with the red border obtained the highest average percentage of user fixation time among all products, and additionally the red border had the greatest declarative impact on the respondents. It is speculated that such a general, somewhat cryptic, highlight may attract more attention than text markings. On the other hand, the ‘eco’ highlight obtained even worse results than products without any highlight, which suggests the high importance of properly selecting the distinctions according to the individual preferences of the prospective user groups. In addition, unsightly, inconsistent colors do not seem to be a problem, rather their subjective perception by the user may be. Some participants paid attention to the navigation bar, indicating this feature was significantly different between the two websites they visited. Moreover, the time spent looking at the navigation bar was longer in the unsightly store. This confirms the importance of navigation for the customers and hence that the lack of aesthetics had limited usefulness to this feature in the study. Despite previous conclusions and regardless of the aesthetics, most people appreciated the first visited page more, and this could be explained by the primacy bias effect.

In view of the above, we suggest applying a few aesthetics-related principles when designing websites in order to enhance the user experience in e-commerce:

- Using the golden ratio rule in photos rather than accidental height-to-width ratios;
- Highlighting product photos by using a border in a color contrasting with the background, rather than using descriptive highlights;
- Ensuring a readable navigation font color distinctive from the background;
- Using consistent spacing between website elements.

We are aware of the limitations of this study, especially of its relatively small sample size. In the future, it would be worth differentiating participants’ demographic factors, such as different age or gender groups, as this may affect the results of aesthetic perception. Additionally, the subject of the study could be extended by a greater number of differences in the online stores’ aesthetics to be compared. Furthermore, we plan to extend the study to mobile devices, due to their growing market share in e-commerce. It would also be interesting to further investigate the impact of aesthetics on augmented and mixed reality applications in the commerce, healthcare, and industrial domains as well [38,39,40]. Overall, the study results proved the benefits of considering the use of aesthetics in the design of online stores so that the most important elements are more noticeable to the customer and will attract their attention in a more efficient way.

## Figures and Tables

**Table 1:** Average fixation time users observed the given AOIs. This time is presented in parentheses as a percentage of the average total fixation time by all users.

	Total	Navigation Bar	Products Grid	Recommendation Area
Aesthetic store	38.5 s	2.4 s (6%)	23.6 s (59.4%)	12.7 s (34.6%)
Unsightly store	38.7 s	2.6 s (6.8%)	25.7 s (64.1%)	10.5 s (29%)

**Table 2:** Average time of fixation on a single product, depending on individual highlight or the lack thereof. This value is given in parentheses as a percentage of the total average viewing time for all products.

	Red Border	Eco	New	None
Aesthetic store	0.81 s (7.97%)	0.65 s (6.42%)	0.68 s (6.72%)	0.65 s (6.42%)
Unsightly store	0.6 s (5.97%)	0.56 s (5.41%)	0.68 s (6.58%)	0.74 s (7.16%)

**Table 3:** Average number of points awarded by participants for each of the highlights.

	Red Border	Eco	New
Average score	2.27	1.57	2.17

## Data Availability

Some data presented in the study are available from the corresponding author upon request, data sharing being limited due to personal information content and the code used during the study being proprietary.
